# Sex Bias in Diagnostic Delay: Are Axial Spondyloarthritis and Ankylosing Spondylitis Still Phantom Diseases in Women? A Systematic Review and Meta-Analysis

**DOI:** 10.3390/jpm14010091

**Published:** 2024-01-13

**Authors:** Francesca Bandinelli, Bianca Martinelli-Consumi, Mirko Manetti, Maria Sole Vallecoccia

**Affiliations:** 1 Rheumatology Department, Usl Tuscany Center, San Giovanni di Dio Hospital, 50143 Florence, Italy; bianca.martinelli1@edu.unifi.it; 2 Section of Anatomy and Histology, Department of Experimental and Clinical Medicine, University of Florence, 50134 Florence, Italy; mirko.manetti@unifi.it; 3 Department of Emergency and Critical Care, Santa Maria Nuova Hospital, 50122 Florence, Italy; mariasole.vallecoccia@uslcentro.toscana.it

**Keywords:** axial spondyloarthritis, spondylitis, delay, sex

## Abstract

Diagnostic delay (DD) is associated with poor radiological and quality of life outcomes in axial spondyloarthritis (ax-SpA) and ankylosing spondylitis (AS). The female (F) population is often misdiagnosed, as classification criteria were previously studied mostly in males (M). We conducted a systematic review to investigate (i) the difference in DD between the sexes, the impact of HLA*B27 and clinical and social factors (work and education) on this gap, and (ii) the possible influence of the year of publication (before and after the 2009 ASAS classification criteria), geographical region (Europe and Israel vs. extra-European countries), sample sources (mono-center vs. multi-center studies), and world bank (WB) economic class on DD in both sexes. We searched, in PubMed and Embase, studies that reported the mean or median DD or the statistical difference in DD between sexes, adding a manual search. Starting from 399 publications, we selected 26 studies (17 from PubMed and Embase, 9 from manual search) that were successively evaluated with the modified Newcastle–Ottawa Scale (m-NOS). The mean DD of 16 high-quality (m-NOS > 4/8) studies, pooled with random-effects meta-analysis, produces results higher in F (1.48, 95% CI 0.83–2.14, *p* < 0.0001) but with significant results at the second analysis only in articles published before the 2009 ASAS classification criteria (0.95, 95% CI 0.05–1.85, *p* < 0.0001) and in extra-European countries (3.16, 95% CI 2.11–4.22, *p* < 0.05). With limited evidence, some studies suggest that DD in F might be positively influenced by HLA*B27 positivity, peripheral involvement, and social factors.

## 1. Introduction

In many rheumatic diseases, the women-to-men ratio is generally considered much higher, and hormones were shown to have a deep influence on disease activity [1,2].

On the other side, ankylosing spondylitis (AS) is a chronic inflammatory disease characterized by progressive disabling axial ankylosing and peripheral involvement, with a higher-than-average incidence in the male (M) population ranging from 2:1 to 3:1 [3,4].

Indeed, we know that estrogens play a role in both enhancing and inhibiting immune reactions, whereas testosterone and progesterone predominantly exert suppressive effects on inflammation [5], though more recently, an implication of sex chromosomes was postulated to explain the links between sex and rheumatic diseases [6].

In recent years, there has been an increasing recognition that AS may have a similar impact on the quality of life in both sexes. Nevertheless, women without a family history of AS were generally underdiagnosed [7].

The disease pattern and severity of AS in the first 10 years of the disease seemed to influence and predict the subsequent natural history [8], and for this reason, an early diagnosis was deemed fundamental [9,10].

Radiological damage is often compounded by diagnostic delay (DD) [11,12], defined as the time between symptoms onset and diagnosis [13], which was found to be much longer in AS [11] than in other rheumatic diseases, such as psoriatic arthritis [14], over the past decades.

After the introduction of the Assessment of Spondyloarthritis International Society (ASAS) classification criteria in 2009 [15], the use of sacroiliac magnetic resonance for early findings of pre-radiographic axial spondyloarthritis (ax-SpA) decreased the DD and the incidence of bamboo spine, thanks to quicker access to treatments [11,16,17,18,19,20,21].

Furthermore, the strong association between the human leukocyte antigen (HLA*)-B27 and spondylitis and its recognition such as the main gene implicated in AS susceptibility [22,23] led to its inclusion in the 2009 ASAS criteria for ax-SpA [23,24]. However, currently, there is no clear consensus on its distribution between female (F) and M sexes, as well as on its impact on DD [14].

Unfortunately, the misdiagnosis of inflammatory back pain [22], the variability of HLA*B27 world distribution, the genetic polymorphism of HLA*B27-negative patients, and cultural prejudices still represent a great obstacle to a fast approach to the diagnosis of AS in women.

This systematic sex bias [23] has unfortunately disadvantaged women, considering this disease’s natural history as equivalent to that in men [22].

While the severity of well-known aspects of spondylitis, such as sacroiliitis [25,26] and lumbar syndesmophytosis [27], seem more manifest in the M population, the cervical spine’s involvement, which is more common in women, is still not included in the classification criteria [27].

In addition, the higher prevalence of peripheral onset in the F population [2,28] might be one of the possible factors of misdiagnosis using SpA classification criteria in women, with an initial correct identification only in 11% vs. 30.2% in men [22].

Based on this background, the aims of the present systematic review were to evaluate (i) the difference in DD between sexes, the impact of HLA*B27 and clinical and social factors (work and education) on this gap, and (ii) the possible influence of the year of publication (before and after the 2009 ASAS classification criteria), region (Europe and Israel vs. extra-European countries), sample sources (mono-center vs. multi-center studies), and world bank (WB) economic class on DD in both sexes.

## 2. Materials and Methods

### 2.1. Search Strategy

We performed a systematic review according to the Preferred Reporting Items for Systematic Reviews and Meta-Analyses (PRISMA) guidelines [29]. This study has not been registered in PROSPERO or any other relevant databases.

We searched in PubMed and Embase following the PRISMA guidelines, namely for titles up to 15th September 2023 regardless of the date of publication, using the following PubMed MeSH terms: (axial spondyloarthritis OR spondylitis) AND (delay) AND (sex). Additionally, the references for relevant articles were hand-searched for the identification of other potentially suitable papers.

This search strategy was designed to include full papers (excluding published conference abstracts, reviews, commentaries, and editorials) that reported differences in DD between M and F sexes. To achieve this goal, exclusion criteria for each item were pre-specified. Letters showing DD prevalence in data for different sexes were considered, but only if they had a sufficiently detailed methodology and results.

### 2.2. Inclusion and Exclusion Criteria

Studies enrolling patients with confirmed ax-SpA and AS, diagnosed with ASAS [15], New York [30], Amor [31], International Statistical Classification of Diseases, Clinical Modification code = 720.0, as listed by the WHO (ICD-9-CM) [32] and ESSG criteria [33], were included; mixed populations of SpA were also included. DD and therapeutic delay were considered as the index test/intervention; F and M sexes were comparators, while the outcomes were HLA*B27, clinical presentation, social parameters, fibromyalgia, and classification criteria.

We excluded studies based only on psoriatic arthritis or those utilizing the same patient cohort of studies already selected as eligible for systematic review.

Considering the possible limits of studies not included in the searchable PubMed and Embase fields, displaying DD in ax-SpA and AS only in the text and not in titles, we performed a forward snowballing of complementary papers with a manual search of cross-references in the citation track [34] for the selected studies and reviews, particularly in previous reports that performed a meta-analysis [14,35].

Studies were included for systematic review if they reported for both sexes the mean or median DD, defined as the difference between the age when the first symptoms of spondylitis were felt (inflammatory back pain using Calin [13,36] or Berlin criteria [15]) or since initial ax-SpA indicators were identified (at least one among other inflammatory axial pain (e.g., cervical spine), peripheral or enthesis symptoms, and uveitis [11]) and the age at AS diagnosis; we also included for the analysis studies that reported both the mean age at onset and at diagnosis [13].

Papers that reported only a statistical analysis of differences in DD were also considered eligible for review, but they were deemed only representative and, therefore, were not included in the final meta-analysis.

### 2.3. Selection Process

**Figure 1:**
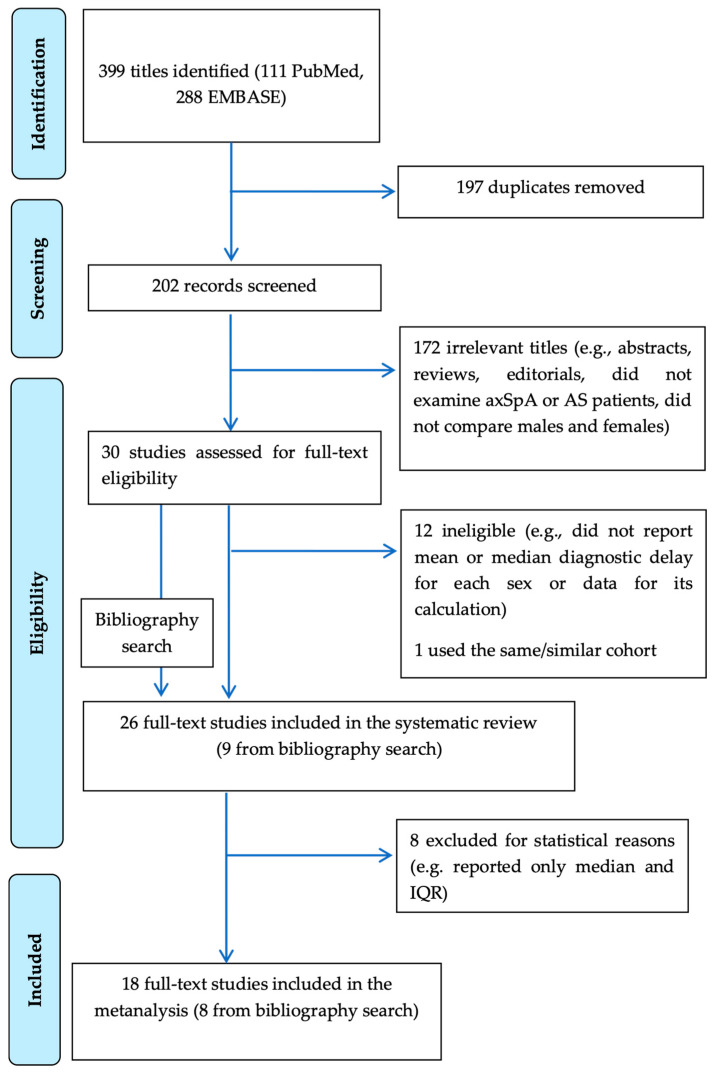
PRISMA flow diagram for the meta-analysis. axSpA, axial spondyloarthritis; AS, ankylosing spondylitis; IQR, interquartile range.

Two reviewers (F.B. and B.M.-C.) independently screened titles and abstracts, assessed full texts for eligibility, and extracted relevant data from qualifying studies. This process adhered to the protocol schedule established from 15 September to 15 November 2023. We followed the PRISMA checklist (see Supplementary Materials); the final PRISMA flow chart is depicted in Figure 1. Any discrepancy at each stage of selection was resolved through discussion moderated by a third reviewer (M.S.V.).

The protocol, including PICO (Patient: AS and ax-Spa; Intervention: DD; Comparator: F vs. M; Outcome: sex) to exclude not eligible articles, was written in accordance with all authors and was not modified in the course of systematic review selection and meta-analysis.

The data of initial eligible studies for systematic review (Figure 1) are shown in Table 1, and information from the included studies was extracted into predefined tabulated summaries as follows: the first author, year of publication, geographic region (similar healthcare systems—Europe and Israel—and extra-European countries), classification criteria for AS and ax-SpA, sample size and sources, F/M ratio, World Bank (WB) economic class [14], mean age at onset (F vs. M), mean age at onset and at diagnosis (F vs. M), mean DD (F vs. M), as well as the significant differences between sexes. We considered Europe and Israel to have similar healthcare systems for the universal right for citizens to access tertiary centers.

Successively, papers were assessed for risk of bias using an adapted version of the modified Newcastle–Ottawa Scale (m-NOS) for case–control studies (see Supplementary Table S1) based on their selection (score 0–4) (disease definition and representativeness), comparability (0–1) of F vs. M, and ascertainment of DD (records 0–1, same method between sexes 0–1, not response rate 0–1 = 0–3).

### 2.4. Statistical Analysis

Finally, we performed a meta-analysis of the pooled mean DD of ax-SpA, AS, and other mixed members of the SpA family, using random-effects models selecting only medium-high quality papers (m-NOS > 4/8). When the mean DD was not reported, it was imputed as the difference in mean age at symptom onset and mean age at diagnosis. When the standard deviation (SD) of DD was missing, we imputed it using the methods recommended by Cochrane as follows: in essence, this was based on the SD of age at onset, the age at diagnosis, and their correlation in all studies or the SD of a study reporting the most similar mean DD duration [61].

Because DD is a continuous variable, the mean difference, its SD, and 95% confidence interval (CI) were used to calculate the global effect of DD by sex; results expressed in the median and interquartile range were excluded.

The meta-analysis was performed using the software ProMeta 3.

Heterogeneity among studies was evaluated using the I^2^ statistics (high heterogeneity if >60% and *p* < 0.1) [62,63]. The effect size was estimated using the unstandardized mean difference reported with its 95% CI. Values of *p* < 0.05 were considered statistically significant. To calculate the pooled effect, a random effect model was applied according to the found heterogeneity (Egger’s linear regression test). Lastly, the funnel plot was visually evaluated to assess possible publication bias.

Following Higgins and Green’s guidelines, which were also successively carried out by a Cochrane review [64], if the heterogeneity of meta-analysis was related to univocal reasons, the study could be removed to produce more confidence in the results [61].

We added the funnel plot to examine the effect sizes estimated from individual studies as a measure of their precision. Furthermore, sensitivity analyses without imputed values were performed to check the stability of the study findings. Specifically, the influence of effect sizes was assessed by the deletion of each paper to check heterogeneous data that could affect the overall results.

We also used random-effects meta-regression to evaluate the influence of the following study characteristics: the year of publication (pre-2009 vs. post-2009), regions with a similar healthcare system (Europe and Israel vs. other extra-European countries), WB (lower-middle vs. upper-middle/high classes) [14], and sample sources (single center vs. multicenter studies). Meta-analysis was not performed for HLA*B27, clinical involvement, and classification criteria, as it was not univocally present in the studies examined, as well as for a number of studies less than 3.

## 3. Results

Starting from a total of 399 publications, we finally selected 26 studies (Figure 1) (15 from PubMed, 2 from Embase, and 9 from manual search) on ax-Spa and AS that reported DD data for both sexes or presented statistical analysis for the difference in DD between M and F populations.

Table 1 shows the age at onset, age at diagnosis, and DD difference between sexes, as well as the main characteristics of the papers (i.e., year of publication, region, sample sources, and WB) and population examined (i.e., classification criteria, sample size, and F/M ratio) in the 26 papers included initially in the systematic review (Figure 1). Collectively, this analysis included a total of 21,704 ax-SpA and AS patients with a total distribution rate of 1:2 for women vs. men (8411 F (38.7%) vs. 13,293 M (61.2%)). The included studies were mostly case–control and cross-sectionally designed, except for one cohort issue [45].

Because of the paucity of studies selected to verify the possible correlation of DD to HLA*B27, clinical aspects, and social parameters (education and work) in AS and ax-SpA, we were not able to conduct any meta-analysis on the studies shown in Table 2 that we described only as a systematic review, also indicating the prevalence of the single parameters examined in both sexes.

### 3.1. HLA*B27

Among the 26 studied eligible for our systematic review, 10 specified the prevalence of HLA*B27 or the difference in its prevalence between sexes, which varied from 55.2% to 94.4% in F and from 75.5% to 98% in M, respectively, as shown in Table 2 [18,22,37,40,44,47,51,52,53,58]. Most of them (6/10) reported a higher prevalence (76.5%–98%) of this haplotype in M [22,40,47,51,53,58] (Table 2). In two of these studies that showed a lower prevalence of HLA*B27 in the F population, DD was generically higher in women compared to men [47,53], whereas other authors did not find a significant difference in DD between sexes [22,40,51,58], even if an exact correlation of HLA*B27 with DD was not specifically analyzed.

Two other studies [37,44] reported no differences in the HLA*B27 distribution between M and F, with a DD that was not significantly different between the two sexes (Table 2).

Only Bandinelli et al. [42] (M 78.02% vs. F 93.18%) and Marks et al. [52] reported a higher prevalence of HLA*B27 in women (M 85.71% vs. F 94.44%) (Table 2).

Regarding the association between the HLA*B27 status and DD, two studies reported a general association between HLA*B27 negativity and longer DD in AS patients, but without distinguishing between men and women: Dincer et al. [46] (5.3 ± 3.5 in HLA*B27 positive vs. 9.2 ± 7.7 in HLA*B27 negative, *p* = 0.037) and Hajialilo et al. [24] (4.6 ± 2.2 in HLA*B27 positive vs. 10.1 ± 3.2 in HLA*B27 negative patients, *p* = 0.0001). Only Bandinelli et al. [42] reported that HLA*B27 positivity was also correlated with a shorter DD in women (Table 2).

### 3.2. Clinical Presentation

Eleven studies variously described the prevalence of joint, enthesis, and spine involvement in the two sexes in ax-SpA and AS at onset or during disease, as shown in Table 2 [22,38,39,40,42,49,51,52,53,58,59].

Peripheral arthritis, whose prevalence varied from 2.5% to 53.3% in women and from 1.2% to 50% in men, respectively, was significantly higher in women in five studies [35,38,40,49,58]; enthesitis in four papers was significantly predominant in women (F 6.8–85.2% vs. M 5.49–80.5%) [35,38,40,42] without significance in other studies (Table 2).

Axial disease was more frequent in M in the lumbar spine (F 15–72% vs. M 34.8–89%) in six papers out of ten that described this aspect [22,38,39,40,51,59]. The prevalence of cervical spine involvement seemed slightly more predominant in women (F 3.7–31.6% vs. M 1.9–10.5%) [22,38,51,52,59], with significant differences only in one paper [38].

Unfortunately, only one paper [42] analyzed the DD related to clinical onset in the two sexes and showed that when axial involvement was the first symptom of ax-SpA and AS, DD might be even higher in men if compared to women with axial or peripheral onset (*p* = 0.0003).

Finally, in six papers, widespread fibromyalgia pain was higher in women (4/6 significantly different) with a prevalence between 13.1% and 50% of cases compared to 0–13.1% in men [35,38,39,42,53,59], but none of them evaluated its role in DD.

### 3.3. Social Parameters

Only six papers investigated the level of education and work of patients (Table 2).

The high level of education varied between studies (in F: 15.9-49.5%, in M: 13.18-54.1%), with controversial results for the comparison of sexes when analyzed in two European papers: Garrido-Cumbrera et al. [47] showed that university education is more common in women, whereas Neuenshwander et al. [53] did not find any difference at all school levels.

Finally, only Bandinelli et al. [42] showed that DD was higher in M with a low level of education (<8 years of education) vs. medium level education (<13 years of education) in F patients (Table 2).

Garrido-Cumbrera et al. [47] and Zink et al. [60] showed that women are employed equally to men, and Neuenschwander et al. [53] reported that they also have similar work absenteeism but with a higher percentage of precarious work (Garrido-Cumbrera et al. [47] and Ogdie et al. [54]).

Blasco-Blasco et al. [43] reported that DD’s effect on work was not significantly different between sexes, whereas another study (Bandinelli et al. [42]) showed that DD in M manual workers was higher than in F non-manual workers.

### 3.4. Meta-Analysis of DD Difference between Sexes

From the initial articles included for systematic review, 8 were excluded for meta-analysis with statistical incoherence as follows: 2 reported only one value of DD for both M and F [41,45], 3 showed only median and interquartile values [22,50,60], and 3 additional papers were excluded due to statistical heterogeneity, as they included subgroups not comparable in the overall analysis of DD differences. Indeed, Blasco-Blasco et al. shared two different values of DD in each sex, one influenced by the effect of the disease on patients’ work life and one regarding its effect on family and partner relationships [43], Ogdie et al. differentiated DD in three periods (<1, 2–9, >10 years) [54], while Ma et al. analyzed data separately from the North and South of China [51].

The final m-NOS, based on the sum of single scores of patient selection, comparability, and DD ascertainment of the 18 articles eligible for meta-analysis, are shown in Supplementary Table S1.

The remaining 18 papers [24,37,38,39,40,42,44,46,47,48,49,52,53,55,56,57,58,59], presenting an m-NOS (0–8) score of 4/8 or more, were cross-sectional case–control studies, 8 mono-centric and 10 multi-centric (only 2 international) studies, published since 1983 to 2023 of which 12 were after and 6 before 2009, 10 were European, and the other 8 were extra-European, 5 were lower-middle WB and 13 were upper-middle or of a high WB class.

The mean value of DD varied from 5.3 and 14.4 in women and from 4.1 and 10.3 in men, respectively, with age at onset (shown only in 9 studies) variable from 23 to 30.4 in F and from 22.2 and 28.3 in M, and age at diagnosis (shown only in 4 papers) variable from 33 to 42.2 in F and from 30.2 and 41.4 in M.

The authors described patients diagnosed with the following criteria: 1/18 New York; 6/18 ASAS; 14/18 modified New York; 3/18 both ASAS and modified New York; 1/18 ESSG criteria in one of the studies that also included ASAS and modified New York (Table 1).

**Figure 2:**
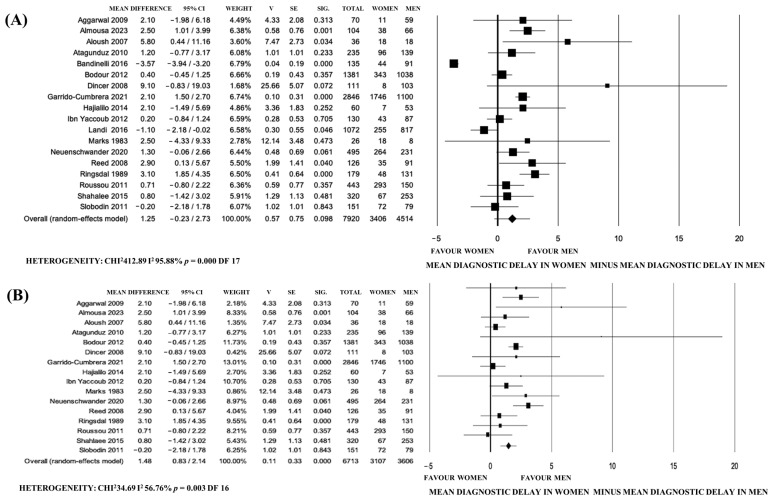
Forest plot of the differences between the mean diagnostic delay of axial spondyloarthritis and spondylitis in women vs. men according to published papers. (**A**) Analysis including 18 papers. (**B**) Analysis including 16 papers.

Eighteen studies were included in the meta-analysis with a high heterogeneity for random-effect meta-analysis (I^2^: 95.88%); for the sensitivity analysis, two recent papers [42,49] presented data dissimilar from other studies (Figure 2A,B).

The first study [42] observed less DD in women than in men, related to higher education, work levels, and peripheral involvement in the F Italian population from two third-level rheumatology centers for early diagnosis. The second [49] was also a recent paper with data from a tertiary hospital and mixed European (Portugal) and South American populations, with ethnic and geographic bias.

**Figure 3:**
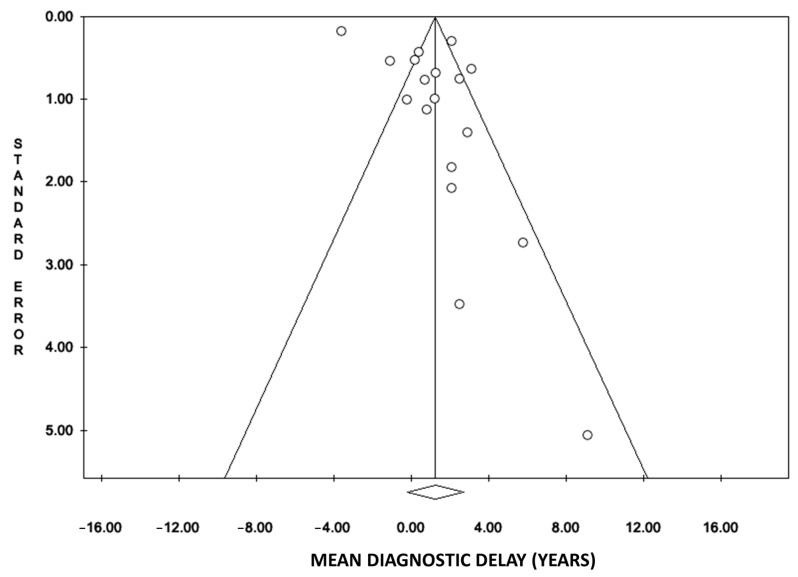
Funnel plot showing publication bias of the published papers on diagnostic delay in women vs. men (18 studies included in the meta-analysis).

The possible publication bias of the 18 studies initially included was confirmed using a funnel plot (Figure 3). The slight asymmetry at the lower left indicates that there is a lack of articles with small samples reporting estimations of lower DD in F compared to M.

Then, a second meta-analysis was carried out on the remaining 16 papers following the Cochrane guidelines [61], which led to the observation of a lower DD in men (a mean difference 1.48 years, 95% CI 0.83–2.14, *p* < 0.0001), with a lower heterogeneity (I^2^: 56.76%) (Figure 2B).

Furthermore, we performed a more detailed meta-analysis based on study characteristics, including namely the year of publication, geographic region (similar healthcare systems -Europe and Israel- vs. extra-European countries), sample sources, and WB economic class.

**Figure 4:**
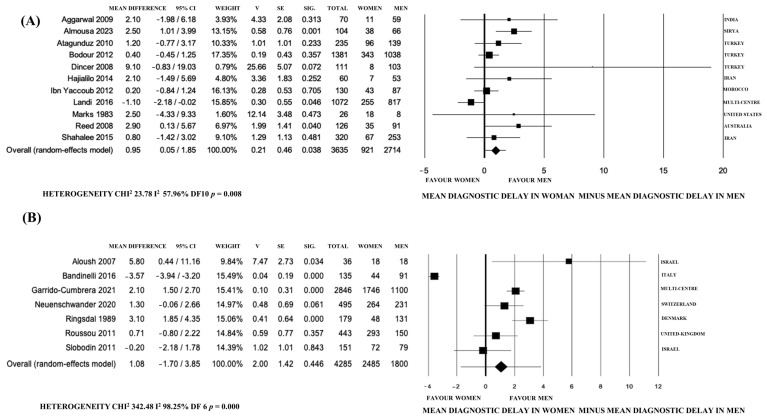
Forest plot of the differences between the mean diagnostic delay of axial spondyloarthritis and spondylitis in women vs. men according to different countries. (**A**) Extra-European countries. (**B**) Europe and Israel. Multi-centric studies were from: Argentina, Brazil, Costa Rica, Chile, Ecuador, Mexico, Peru, Uruguay, and Portugal (**A**); Austria, Belgium, France, Germany, Italy, Netherlands, Norway, Russia, Slovenia, Spain, Sweden, Switzerland and the United Kingdom (**B**).

**Figure 5:**
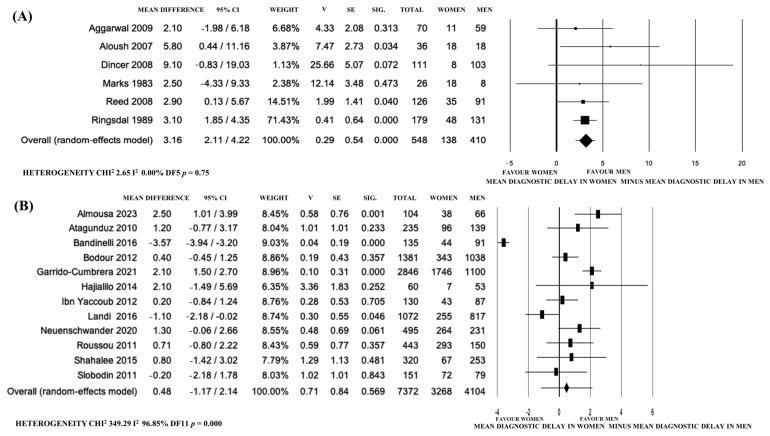
Forest plot of the differences between the mean diagnostic delay of axial spondyloarthritis and spondylitis in women versus men according to the year of publication. (**A**) Papers published before 2009. (**B**) Papers published after 2009.

The studies published before 2009 showed higher DD in F (mean difference 3.16 years, 95% CI 2.11–4.22, *p* < 0.0001, I^2^: 0%) and in non-European countries (mean difference 0.95 years, 95% CI 0.05–1.85, *p* = 0.04, I^2^: 57.96%, *p* = 0.008), while after 2009 and in studies from similar healthcare systems of Europe and Israel, DD was similar (Figure 4 and Figure 5).

Sample sources (mono-centric vs. multi-centric papers) and WB class seemed not to influence DD in the meta-analysis, as shown in Supplementary Figures S1 and S2.

Because of the non-univocal presence of classification criteria in these studies, a sub-analysis of ASAS classification criteria was not possible.

## 4. Discussion

Our systematic review showed that DD in ax-SpA and AS was higher in women. Even if the final meta-analysis considered only limited descriptive cross-sectional studies eligible, reporting large samples only in a few cases, our analysis did not show significant differences in sex-related DD between mono-centric and more representative multi-centric studies.

Furthermore, to reduce the initial heterogeneity and publication bias of meta-analysis, we had to exclude, in the final sensitivity analysis, two recent papers from third-level centers. In particular, the first one [42] observed a lower DD in F than in M related to higher education, work levels, and peripheral involvement; the second one [49] also had geographic and ethnic bias, focusing on patients from Europe (Portugal) and South America.

We know that the disease is often not suspected in the F population, leading to an incorrect or nonspecific diagnosis in healthcare records: Jovani et al. [22] reported that only 11% of F received a correct diagnosis vs. 30% of M, and also that F had a number of specialist visits that was significantly higher than M.

We also know that DD might have important consequences in terms of radiological progression [11], and the difference in spinal progression observed in patients is also depicted in relation to symptom duration.

Commonly, women are considered less prone to develop a disability, and the difference in DD between sexes was attributed to poorer spinal mobility and radiological damage in men who consent to an earlier diagnosis [35,48]. This means that women might have difficult conditions for a long time, continuously looking for the right diagnosis and treatment without a solution [22].

Otherwise, in F, the self-ratings of handicap scores were generally worse than in M, and pain intensity increased with age [60].

The lower prevalence of HLA*B27 in F [22,40,47,51,53,58], reported in most of the papers analyzed, might also impact the diagnosis, as demonstrated only by Bandinelli et al. [42], who found a lower DD in HLA*B27-positive women.

We can assume that, as proposed by Hajialilo [24], since HLA*B27 was introduced as a criterion for the diagnosis of AS patients in the ASAS classification criteria, physicians visit HLA*B27-positive patients earlier, facilitating the diagnosis.

The difference in the gender distribution of HLA*B27 in the studies examined is difficult to interpret: HLA*B27 belongs to a family of closely related cell-surface proteins encoded in the HLA*B locus, located within chromosome 6, apparently without any known sex-related hereditary [65].

The over-estimation of HLA*B27-negative women in published studies might be a consequence of an incorrect initial referral lacking genetic and familial investigation for SpA and the inclusion of F in the study population only after unsuccessful treatment under a different diagnosis [58].

In addition, even if substantial evidence exists that HLA*B27 might have a direct role in genetic susceptibility to AS/SpA, HLA*B27 is only responsible for ~20% of the overall genetic risk [66]; the underlying molecular basis has yet to be identified, and it might not be a prerequisite for the occurrence of AS since this disease also affects individuals who lack this gene [65,67].

Interestingly, in some geographic areas, newly generated *B27 subtypes might include protective haplotypes, such as *B27 09 in Sardinia [68] and *B27 06 in Southeast Asia [69], offering a novel perspective to dissect disease pathogenesis and identify additional genetic factors of susceptibility [70].

Furthermore, there are numerous new genetic loci that are known to be associated with increased risk for AS ([71,72]), and the possibility that AS susceptibility could be related to other MHC genes is increasing [73]. For instance, in the Portuguese population, the genotype HLA*-F01 showed a possible susceptibility effect, while other F and G haplotypes seemed to be protective [74].

Thus, in future studies, other haplotypes might be more deeply studied and included in the clinical classification of the F AS and ax-SpA populations.

Secondly, another possible explanation for DD bias between sexes, previously hypothesized by Jovani et al. [35], might be the different prevalence in clinical symptoms at the onset or during the disease that might delay the diagnosis, which was confirmed by our systematic review that found a higher prevalence of joint, enthesis, and cervical spine involvement in women [35,38,40,49,58] or fibromyalgia overlap [38,39,53,54,59]. Otherwise, only one study analyzed the correlation of clinical presentation at the onset with DD [42], showing that when axial involvement is the first symptom of the disease, DD might be even higher in M than in F.

In the F population, some symptoms of SpA, like morning stiffness and sleep disorders associated with diffuse enthesis pain, are frequently not interpreted in the same way as the M population. These symptoms might be mistakenly associated with fibromyalgia or incorrectly referred to surgeons or internal specialists [59] or even psychiatry [75,76].

Finally, our meta-analysis showed that DD was more pronounced in F in papers published in extra-European countries and before 2009.

The high significance and low heterogeneity of the analysis of studies published before 2009 may be due to the diffuse use of the New York criteria in the past [37,39,56], which was deemed essential for the presence of radiographic sacroiliitis and were validated prevalently in the M population [30]. In fact, the new ASAS classification criteria were validated in 2009, and successively, a pelvis MRI and evaluation of peripheral involvement were introduced in clinical practice for AS and ax-SpA diagnosis [15].

Nevertheless, the ASAS classification criteria might also present some black holes. Firstly, the involvement of the cervical spine seemed higher in women [38], as was also confirmed in a recently published article [27] that showed a slightly higher number of cervical syndesmophytes [27,77] compared to lumbar ankylosis in F, which is a phenomenon that was not observed in men.

Otherwise, we should consider the potential limits of our current analysis. Firstly, the quality of the research on SpA has increased over the last twenty years, and old studies might have methodological limits due to limited sample size and possible selection bias. Indeed, the predominant source of information in the past decade has often been hospitals, which might be less accessible through primary healthcare channels. In addition, the year of publication was the only available representation of change in the diagnostic approach because a precise comparison between different classification criteria was not possible, as it is often contemporaneously present in studies, even though, as shown by Zhao et al. [14] the intervals between recruitment and publication are generally homogeneous in different studies.

The higher DD in women in the extra-European countries could be explained by the difference in healthcare system compared to Israel and Europe. Indeed, in these regions, citizens have the right to universal healthcare and access to tertiary centers for early diagnosis through MRI and HLA testing.

Another possible explanation for such a different DD distribution between sexes might be the similar genetic distribution, given that most of the Israeli population migrated from Europe after the Second World War. In fact, it was observed that the strength of this association of HLA*B27 varies among some of the ethnic and racial groups in the world [65,69,78]. Furthermore, the geographic distribution of HLA*B27 shows a latitude-related gradient inverse to that of the malaria endemic, with an apparent exception in New Guinea, a region where malaria is present, but where HLA*B27 frequency shows, an orographic gradient antithetic to the malaria incidence [79,80].

Otherwise, in our study, genetic distribution was necessarily approximated to great geographic areas, and a more specific ethnic analysis might better clarify the different distribution between genders in the future, together with an analysis of gene polymorphisms.

## 5. Conclusions

This article highlights a fascinating and actual theme of gender bias in ax-SpA and AS, showing a poorer DD in the F population through a systematic review and meta-analysis of the literature from the last 40 years, from 1983 to 2023.

This research aimed to evaluate the difference in DD between sexes and the impact of genetic (HLA*B27), clinical conditions, education, and work factors on this gap, and the possible influence of the year and sample source of publications, geographic region, and WB economic class on DD in both sexes.

The different prevalence of HLA* B27 positivity, clinical presentation (peripheral, enthesis, and cervical spine involvement), education, and manual work between sexes might be an influence on DD, as shown only by limited evidence not sufficient for meta-analysis. For these reasons, future research is deemed essential, especially on genetic aspects and more modern and tailored ax-Spa and AS criteria to avoid the serious and important consequences of sex bias still present in the clinical management of women’s health.

Moreover, in the second part of our meta-analysis, we argued that the year of publication and the geographic provenience might have a deep impact on DD. In fact, studies performed before 2009 and from extra-European countries reported higher DD.

We might conclude that the increase in the quality of the research on ax-SpA and AS in recent years and the use of the ASAS classification criteria has improved the operative definition of disease in different genders. At the same time, the research approach still risks decontextualizing women’s health and under-sizing the sex bias, as a consequence of a skein of responsibilities still difficult to unravel.

## Figures and Tables

**Table 1:** Summary of the main characteristics of the studies on DD difference between sexes, with data expressed as the mean (SD) or median (IQR, interquartile range) (*), included in the systematic review (*n* = 26) and in the meta-analysis (*n* = 18). Articles in bold were included in the meta-analysis.

Study	Disease	Classification Criteria	Sample Size and F:M Ratio	Sample Source	WB Class	Mean Age at Symptom Onset F vs. M	Mean Age at Diagnosis F vs. M	Mean Delay to Diagnosis F vs. M
**Aggarwal 2009** [37] ***IND***	AS	mNY	70 11:59	SC	Lower-middle	NR	NR	8.6 (6.6) vs. 6.5 (4.7) *p* = 0.2
**Almousa 2023** [38] ***SYR***	ax-Spa	ASAS	114 38:76	MC	Lower-middle	25.0 (5.1) vs. 25.1 (6.1) *p* = 0.9	NR	9.2 (3.9) vs. 6.7 (3.4) *p* = 0.001
**Aloush 2007** [39] ***ISR***	AS	mNY	36 18:18	SC	High	NR	NR	9.9 vs. 4.1 *p* = 0.05
**Atagunduz 2010** [40] ***TUR***	AS	mNY	235 96:139	MC	Upper-middle	29.7 (10.2) vs. 25.1 (9.0) *p* = 0.87	NR	7.4 (7.9) vs. 6.2 (7.1) *p* = 0.8
Bakland 2011 [41] *EUR*	AS	mNY	677 166:511	SC	High	NA	NR	*p* = NS
**Bandinelli 2016** [42] ***EUR***	ax-SpA	ASAS/ mNY	135 44:91	SC	High	NA	NA	6.3 (1.1) vs. 9.9 (0.8) *p* = 0.002
Blasco-Blasco 2017 [43] *EUR*	SpA	Self-reported ASAS	145 52:93	SC	High	NR	NR	*p* = 0.1 (in patients with DD impact on work)
**Bodur 2012** [44] ***TUR***	AS	mNY	1381 343:1038	NT	Upper-middle	30.4 (10.0) vs. 26.5 (9.5) *p* = 0.000	NA	5.3 (7.0) vs. 4.9 (6.9) *p* = 0.4
Deodhar 2016 [45] *USA*	AS	ICD-9-CM	3336 1677: 1659	NT	High	NR	NR	NR *p* = 0.016
**Dincer 2008** [46] ***TUR***	AS	mNY	111 8:103	SC	Upper-middle	NA	NA	14.4 (14.2) vs. 5.3 (5.7) *p* = 0.06
**Garrido-** **Cumbrera 2021** [47] ***EUR***	ax-SpA	Self- reported ASAS	2846 1746: 1100	MC	High	26.4 (10.7) vs. 27.0 (11.8) *p* = 0.3	34.4 (10.9) vs. 32.6 (12.2) *p* < 0.001	8.2 (8.9) vs. 6.1 (7.4) *p* < 0.001
**Hajialilo 2014** [24] ***IRN***	AS	mNY	60 7:53	SC	Upper-middle	NR	NA	8.0 (4.7) vs. 5.9 (3.3) *p* = 0.14
**Ibn Yacoub 2012** [48] ***MAR***	AS	mNY	130 43:87	SC	Lower-middle	28.8 (10.7) vs. 27.9 (11.1) *p* = 0.4	NR	4.8 (2.7) vs. 4.6 (3.1) *p* = 0.07
Jovani 2018 [22] *EUR*	ax-SpA and AS	ASAS/ mNY/ ESSG/ Amor	150 54:96	SC	High	31.2 vs. 30.9 *p* = 0.9	41.9 vs. 39.1 *p* = 0.2	* 7.5 (11) vs. * 4 (11) *p* = 0.05
**Landi 2016** [49] ***SA***	AS	mNY	1072 255:817	MC	Upper-middle	NR	NR	7.8 (7.5) vs. 8.9 (8.2) *p* = 0.1
Li 2019 [50] *CHN*	ax-SpA and AS	ASAS, mNY	208 59:149	SC	Upper-middle	NA	NR	* 12.5 (1.5, 56.0) vs. * 35.0 (5.5, 87.3) (months) *p* = 0.014
Ma 2012 [51] *CHN*	AS	mNY	150 36:114	MC	Upper-middle	south: 26.6 (6.6) vs. 24.1 (6.9); north: 28.6 (9.4) Vs. 26.9 (10.9)	NR	south: 6.2 (6.4) vs. 6.6 (6.0); north: 4.1 (6.3) vs. 4.0 (5.2)
**Marks 1983** [52] ***USA***	AS	NY	50 25:25	SC	High	23 vs. 22.2	NR	12.8 vs. 10.3
**Neuenschwander** **2020** [53] ***EUR***	ax-SpA	ASAS	495 264:231	SC	High	28.7 (9.1) vs. 28.3 (8.4) *p* = 0.6	NR	6.0 (7.8) vs. 4.7 (7.6) *p* = 0.005
Ogdie 2019 [54] *USA*	AS	Self reported mNY	235 174:61	MC	High	NR	NR	NR
**Reed 2008** [55] ***AUS***	AS	mNY	126 35:91	SC	High	NA	NA	10.2 vs. 7.3
**Ringsdal 1989** [56] ***EUR***	AS	Self-reported mNY	179 48:131	SC	High	NA	NR	12.6 vs. 9.5
**Roussou 2011** [57] ***EUR***	ax-SpA and AS	ASAS/ mNY /ESSG	516 344:172	SC	High	NR	42.2 (13.8) vs. 41.4 (14.9) *p* = NS	6.3 (7.2) vs. 5.6 (7.9) *p* = NS
**Shahlaee A 2015** [58] ***IRN***	AS	mNY	320 67:253	NT	Upper-middle	24.3 (7.73) vs. 22.2 (7.14) *p* = 0.04	33 (9.5) vs. 30.2 (8.9) *p* = 0.025	8.8 (8.5) vs. 8 (7.2) *p* = 0.5
**Slobodin 2011** [59] ***ISR***	ax-SpA and AS	ASAS/ mNY	151 72:79	MC	High	NR	38.5 (12.3) vs. 35.6 (11.7) *p* = 0.13	5.7 (6.0) vs. 5.9 (6.4) *p* = 0.9
Zink 2000 [60] *EUR*	AS	mNY	8776 2729: 6047	NT	High	29.1 (11.4) vs. 27.9 (10.9)	NR	6.1 (8.1) vs. 5.5 (7.5)

Abbreviations: ax-SpA, axial spondyloarthritis; ASAS, Assessment of Spondyloarthritis international Society classification criteria; AS, ankylosing spondylitis; AUS, Australia; CHN, China; ESSG, European Spondyloarthropathy Study Group criteria; EU, Europe; ISR, Israel; F, females; ICD-9-CM, International Classification of Diseases, Ninth Revision, Clinical Modification; IND, India; IRN, Iran; SA, South America; M, males; MAR, Marocco; MC, multi-center; mNY, modified New York criteria; NA, not applicable because the reported data made no distinction between females and males; NR, not reported; NS, not significant; NT, national; NY, New York criteria; SC, single-center; SpA, spondyloarthritis; SYR, Syria; TUR, Turkey; USA, United States of America; WB, World Bank economic class.

**Table 2:** Studies analyzed only in the systematic review that included HLA*B27, peripheral, enthesis, axial, and fibromyalgia clinical presentation, social factors (work and education) prevalence, and its possible influence on DD, with data expressed as the percentage (%), mean ± standard deviation (SD) or median ± interquartile range (IQR).

Study	HLA*B27, Clinical Presentation, Prevalence of Social Factors, and Their Influence on DD
Aggarwal et al. 2009 [37]	▪ HLA*B27+, M vs. F, %: no difference in distribution between sexes ▪ DD, F vs. M, mean ± SD: 8.6 ± 6.6 vs. 6.5 ± 4.7, *p* = 0.23
Almousa et al. 2023 [38]	▪ Fibromyalgia syndrome at onset, F vs. M, %: 15.8 vs. 2.6, *p* = 0.01 ▪ Clinical presentation at onset, F vs. M, %: arthritis 38.8 vs. 14.5, *p* = 0.007; low back pain 23.7 vs. 69.7, *p* < 0.001; enthesitis 28.9 vs. 15.8, *p* = 0.09; uveitis 5.3 vs. 6.6, *p* = 0.7; widespread pain 50 vs. 21, *p* = 0.002; psoriasis 5.3 vs. 0
Aloush et al. 2007 [39]	▪ Fibromyalgia, F vs. M, %: 50 vs. 0, *p* = 0.0002 ▪ Enthesitis, F vs. M, mean: 12.2 vs. 5.8, *p* = 0.008
Atagunduz et al. 2010 [40]	▪ HLA*B27+, M vs. F, %: 75.5 vs. 63.3, *p* = 0.05 ▪ DD, F vs. M, mean ± SD: 7.4 ± 7.9 vs. 6.2 ± 7.1, *p* = 0.78 ▪ Clinical presentation, F vs. M, %: peripheral arthritis 53.3 vs. 30.9, *p* = 0.001; enthesitis 64.8 vs. 36.4, *p* = 0.001; hip involvement 52 vs. 50, *p* = 0.65; uveitis 26.2 vs. 20.6, *p* = 0.09; early axial ankylosing 15 vs. 34.8, *p* = 0.001
Bandinelli et al. 2016 [42]	▪ HLA*B27+, M vs. F, %: 78.02 vs. 93.18 ▪ DD, F vs. M, mean ± SD: 6.3 ±1.1 vs. 9.9 ± 0.8, *p* = 0.0023 ▪ DD in HLA*B27+ vs. HLA*B27-: no significant difference ▪ Manual work, F vs. M, %: 6.81 vs. 27.47 ▪ DD in M manual workers vs. in F non-manual workers, mean ± SD: 10.7 years ± 1.4 vs. 6.4 ± 1.2, *p* = 0.0186 ▪ DD in M with a low level of education vs. in F with a medium level of education, mean ± SD: 11.4 ± 1.4 years vs. 6.5 ± 1.4, *p* = 0.0045 ▪ Clinical onset, M vs. F, %: inflammatory back pain 84.6 vs. 93.18, peripheral arthritis 16.48 vs. 29.54, enthesitis 5.49 vs. 6.81 ▪ Comparison of DD with axial involvement as the first symptom, M vs. F, mean ± SD: 10.3 ± 0.9 vs. 6.6 ± 1.2, *p* = 0.0003
Blasco-Blasco et al. 2017 [43]	▪ Manual work in F vs. M, %: 60 vs. 77.6, *p* = 0.09 ▪ DD effect on work life, F vs. M, yrs, mean (95% CI): 12.8 (7.9–17.8) vs. 8.4 (5.9–10.9), *p* = 0.1
Bodur et al. 2012 [44]	▪ HLA*B27+, M vs. F, %: no significant difference between sexes ▪ DD, F vs. M, mean ± SD: 5.3 ± 7.0 vs. 4.9 ± 6.9, *p* = 0.385
Dincer et al. 2008 [46]	▪ DD in HLA*B27+ vs. HLA*B27-, mean ± SD: 5.3 ± 3.5 vs. 9.2 ± 7.7, *p* = 0.037
Garrido-Cumbrera et al. 2021 [47]	▪ HLA*B27+, M vs. F, %: 80.2 vs. 66.7, *p* < 0.001 ▪ DD, F vs. M, mean ± SD: 8.2 ± 8.9 vs. 6.1 ± 7.4, *p* < 0.001 ▪ Employed F vs. M, %: 87 vs. 89.2, *p* = 0.125 ▪ F in temporary sick leave vs. M, %: 33.8 vs. 21.6, *p* < 0.001 ▪ University level of education in F vs. M, %: 49.5 vs. 45.9, *p <* 0.001
Hajialilo 2014 [24]	▪ DD in HLA*B27+ vs. HLA*B27-, mean ± SD: 4.6 ± 2.2 vs. 10.1 ± 3.2, *p* = 0.0001
Jovani et al. 2018 [22]	▪ HLA*B27+ M vs. F, %: 77.1 vs. 59.3, *p* = 0.02 ▪ DD, F vs. M, median (IQR): 7.5 (11.5) vs. 4 (11), *p* = 0.053 ▪ Clinical presentation at onset, F vs. M, %: according to patients: back pain 61.5 vs. 74.5, *p* = 0.10; peripheral involvement 57.7 vs. 35.2, *p* = 0.008. According to medical records: back pain was 44.4 vs. 82.1, *p* < 0.001; peripheral involvement was 55.6 vs. 17.9, *p* < 0.001
Landi et al. 2016 [49]	▪ Clinical presentation, F vs. M, %: uveitis/iritis 23.4 vs. 23.9, *p* = 0.47; dactilitis 7.9 vs. 8.5, *p* = 0.44; enthesitis 67.9 vs. 41.1, *p* < 0.001
Ma et al. 2012 [51]	▪ HLA*B27+, M vs. F, %: southern China: 98 vs. 89.5; northern China: 87.3 vs. 82.3 ▪ DD, F vs. M, mean ± SD: southern China: 6.2 ± 6.4 vs. 6.6 ± 6.0; northern China: 4.1 ± 6.3 vs. 4.0 ± 5.2 ▪ Symptoms at onset, F vs. M, %: southern China: inflammatory spinal pain 52.6 vs. 64.7, neck pain 10.5 vs. 1.9, low back pain 31.5 vs. 54.9, peripheral arthritis 15.7 vs. 15.6; northern China: inflammatory spinal pain 58.8 vs. 66.6, neck pain 5.8 vs. 1.5, low back pain 47.05 vs. 52.3, peripheral arthritis 29.4 vs. 22.2
Marks et al. 1983 [52]	▪ HLA*B27+, M vs. F, %: 85.7 vs. 94.4 ▪ Clinical presentation, F vs. M (adult onset AS), %: cervical arthritis 35 vs. 24, shoulder disease 35 vs. 18, hip disease 24 vs. 18
Neuenschwander et al. 2020 [53]	▪ HLA*B27+, M vs. F, %: 76.5 vs. 67, *p* = 0.03 ▪ DD, F vs. M, mean ± SD: 6.0 ± 7.8 vs. 4.7 ± 7.6, *p* = 0.005 ▪ Fibromyalgia, F vs. M, %: 13.1 vs. 2.7, *p* < 0.001 ▪ Abstenteism within last year in F vs. M, %: 44 vs. 53.9, *p* = 0.12 ▪ University level of education, F vs. M, %: 28.3 vs. 27.4 ▪ Clinical characteristics of nr-ax-SpA patients, F vs. M, %: enthesitis 79.6 vs. 64, *p* < 0.001; peripheral arthritis 39.2 vs. 35.8, *p* = 0.46
Ogdie et al. 2019 [54]	▪ Part-time employed F vs. M, %: 9.2 vs. 4.9 ▪ Fibromyalgia, F vs. M, %: 43.7 vs. 13.1, *p* < 0.05
Shahlaee et al. 2015 [58]	▪ HLA*B27+ M vs. F, %: 78.3 vs. 55.2, *p* < 0.001 ▪ DD, F vs. M, mean ± SD: 8.8 ± 8.5 vs. 8.0 ± 7.2, *p* = 0.464 ▪ Clinical characteristics of AS patients, F vs. M, %: enthesitis 82.1 vs. 68.8, *p* = 0.032; elbow joint involvement 9 vs. 2.8, *p* = 0.023
Slobodin et al. 2011 [59]	▪ Fibromyalgia-like generalized pain at diagnosis, F vs. M, %: 39 vs. 6.3, *p* < 0.0001 ▪ Presenting symptom, F vs. M, %: inflammatory low back pain 73 vs. 89, *p* = 0.02; neck pain 11 vs. 5, *p* = 0.23; knee arthritis 11 vs. 14, *p* = 0.6; uveitis 7 vs. 5, *p* = 0.74; heel pain 7 vs. 1.3, *p* = 0.23
Zink et al. 2000 [60]	▪ Employed F vs. M, %: 62.5 vs. 75.3

Abbreviations: ax-SpA, axial spondyloarthritis; AS, ankylosing spondylitis; CI, confidence interval; DD, diagnostic delay; F, females; HLA*, human leukocyte antigen; IQR, interquartile range; M, males; nr-ax-SpA, non-radiographic ax-SpA; SD, standard deviation.

## Data Availability

Data and protocol are available if requested.

## Supplementary Materials

The following supporting information can be downloaded at: https://www.mdpi.com/article/10.3390/jpm14010091/s1, Table S1: m-NOS quality assessment of 26 studies eligible for systematic review; Figure S1: Forest plot of the differences between the mean diagnosis delay of ax-SpA and AS in women versus men according to World Bank economic class in (A) papers from low and lower-middle income countries and (B) papers from upper-middle- and high-income countries; Figure S2: Forest plot of the differences between the mean diagnostic delay of ax-SpA and AS in women vs. men according to samples of (A) mono-center and (B) multi-center studies; PRISMA checklist.

## References

[1] Cutolo M., Straub R.H. (2020). Sex Steroids and Autoimmune Rheumatic Diseases: State of the Art. Nat. Rev. Rheumatol..

[2] Chimenti M.-S., Alten R., D’Agostino M.-A., Gremese E., Kiltz U., Lubrano E., Moreno M., Pham T., Ramonda R., Spinelli F.-R. (2021). Sex-Associated and Gender-Associated Differences in the Diagnosis and Management of Axial Spondyloarthritis: Addressing the Unmet Needs of Female Patients. RMD Open.

[3] Wright K.A., Crowson C.S., Michet C.J., Matteson E.L. (2015). Time Trends in Incidence, Clinical Features, and Cardiovascular Disease in Ankylosing Spondylitis over Three Decades: A Population-Based Study. Arthritis Care Res..

[4] Salaffi F., Siragusano C., Alciati A., Cassone G., D’Angelo S., Guiducci S., Favalli E.G., Conti F., Gremese E., Iannone F. (2022). Axial Spondyloarthritis: Reshape the Future-From the “2022 GISEA International Symposium”. J. Clin. Med..

[5] Benagiano M., Bianchi P., D’Elios M.M., Brosens I., Benagiano G. (2019). Autoimmune Diseases: Role of Steroid Hormones. Best Pract. Res. Clin. Obstet. Gynaecol..

[6] Lambert N.C. (2019). Nonendocrine Mechanisms of Sex Bias in Rheumatic Diseases. Nat. Rev. Rheumatol..

[7] Feldtkeller E., Braun J. (2000). Impact of Sex on Inheritance of Ankylosing Spondylitis. Lancet.

[8] Pradeep D.J., Keat A., Gaffney K. (2008). Predicting Outcome in Ankylosing Spondylitis. Rheumatology.

[9] Diaconu A.-D., Ceasovschih A., Șorodoc V., Pomîrleanu C., Lionte C., Șorodoc L., Ancuța C. (2022). Practical Significance of Biomarkers in Axial Spondyloarthritis: Updates on Diagnosis, Disease Activity, and Prognosis. Int. J. Mol. Sci..

[10] Adshead R., Donnelly S., Knight P., Tahir H. (2020). Axial Spondyloarthritis: Overcoming the Barriers to Early Diagnosis-an Early Inflammatory Back Pain Service. Curr. Rheumatol. Rep..

[11] Salvadorini G., Bandinelli F., Delle Sedie A., Riente L., Candelieri A., Generini S., Possemato N., Bombardieri S., Matucci-Cerinic M. (2012). Ankylosing Spondylitis: How Diagnostic and Therapeutic Delay Have Changed over the Last Six Decades. Clin. Exp. Rheumatol. Incl. Suppl..

[12] Neto A., Figueira R., Quintal A., Rodrigues M. (2021). The Natural Progression of Ankylosing Spondylitis After 4 Decades of Untreated Disease. J. Clin. Rheumatol..

[13] Feldtkeller E., Erlendsson J. (2008). Definition of Disease Duration in Ankylosing Spondylitis. Rheumatol. Int..

[14] Zhao S.S., Pittam B., Harrison N.L., Ahmed A.E., Goodson N.J., Hughes D.M. (2021). Diagnostic Delay in Axial Spondyloarthritis: A Systematic Review and Meta-Analysis. Rheumatology.

[15] Sieper J., van der Heijde D., Landewe R., Brandt J., Burgos-Vagas R., Collantes-Estevez E., Dijkmans B., Dougados M., Khan M., Leirisalo-Repo M. (2009). New Criteria for Inflammatory Back Pain in Patients with Chronic Back Pain: A Real Patient Exercise by Experts from the Assessment of SpondyloArthritis International Society (ASAS). Ann. Rheum. Dis..

[16] Bandinelli F., Delle Sedie A., Salvadorini G., Riente L., Candelieri A., Generini S., Bombardieri S. (2013). Reply to Comment on ‘Ankylosing Spondylitis: How Diagnostic and Therapeutic Delay Have Changed over the Last Six Decades’ E. Feldtkeller, A. Zeller, M. Rudwaleit. Clin. Exp. Rheumatol..

[17] Bandinelli F., Terenzi R., Giovannini L., Milla M., Genise S., Bagnoli S., Biagini S., Annese V., Matucci-Cerinic M. (2014). Occult Radiological Sacroiliac Abnormalities in Patients with Inflammatory Bowel Disease Who Do Not Present Signs or Symptoms of Axial Spondylitis. Clin. Exp. Rheumatol..

[18] Bandinelli F., Melchiorre D., Scazzariello F., Candelieri A., Conforti D., Matucci-Cerinic M. (2013). Clinical and Radiological Evaluation of Sacroiliac Joints Compared with Ultrasound Examination in Early Spondyloarthritis. Rheumatology.

[19] Tas N.P., Kaya O., Macin G., Tasci B., Dogan S., Tuncer T. (2023). ASNET: A Novel AI Framework for Accurate Ankylosing Spondylitis Diagnosis from MRI. Biomedicines.

[20] Benucci M., Damiani A., Bandinelli F., Russo E., Li Gobbi F., Grossi V., Amedei A., Infantino M., Manfredi M. (2022). Correction: Benucci et al. Predicting Loss of Efficacy after Non-Medical Switching: Correlation between Circulating TNF-α Levels and SB4 in Etanercept to SB4 Switchers and Naïve Patients with Rheumatic Disease. J. Pers. Med..

[21] Favalli E.G., Becciolini A., Caporali R., Todoerti M., Iannone F., Dinoia L., Sebastiani M., Spinella A., Gremese E., Cianci F. (2018). The Profiling of Axial Spondyloarthritis Patient Candidate to a Biologic Therapy: Consensus from a Delphi-Panel of Italian Experts. Autoimmun. Rev..

[22] Jovani V., Blasco-Blasco M., Pascual E., Ruiz-Cantero M.T. (2018). Challenges to Conquer from the Gender Perspective in Medicine: The Case of Spondyloarthritis. PLoS ONE.

[23] Chaudhary H., López-Medina C., Khan M.A., Dougados M., Magrey M. (2023). Clinical Profile and Treatment Utilisation Based on HLA-B*27 Status in Axial Spondyloarthritis: Results from ASAS-PerSpA Study. RMD Open.

[24] Hajialilo M., Ghorbanihaghjo A., Khabbazi A., Kolahi S., Rashtchizadeh N. (2014). Ankylosing Spondylitis in Iran; Late Diagnosis and Its Causes. Iran. Red. Crescent Med. J..

[25] Xiong J., Chen J., Tu J., Ye W., Zhang Z., Liu Q., Zhu X. (2014). Association of HLA-B27 Status and Gender with Sacroiliitis in Patients with Ankylosing Spondylitis. Pak. J. Med. Sci..

[26] Min H.K., Cho H., Park S.-H. (2019). Baseline Severity of Sacroiliitis Can Predict Acute Inflammatory Status of Sacroiliac Joint in Early Axial Spondyloarthritis of Male Patients: A Cross Sectional Study. BMC Musculoskelet. Disord..

[27] Ensslin C., Micheroli R., Kissling S., Götschi A., Bürki K., Bräm R., de Hooge M., Baraliakos X., Nissen M.J., Möller B. (2023). Impact of Sex on Spinal Radiographic Progression in Axial Spondyloarthritis: A Longitudinal Swiss Cohort Analysis over a Period of 10 Years. RMD Open.

[28] Bandinelli F., Manetti M., Ibba-Manneschi L. (2016). Occult Spondyloarthritis in Inflammatory Bowel Disease. Clin. Rheumatol..

[29] Page M.J., McKenzie J.E., Bossuyt P.M., Boutron I., Hoffmann T.C., Mulrow C.D., Shamseer L., Tetzlaff J.M., Akl E.A., Brennan S.E. (2021). The PRISMA 2020 Statement: An Updated Guideline for Reporting Systematic Reviews. BMJ.

[30] van der Linden S., Valkenburg H.A., Cats A. (1984). Evaluation of Diagnostic Criteria for Ankylosing Spondylitis. A Proposal for Modification of the New York Criteria. Arthritis Rheum..

[31] Amor B., Dougados M., Mijiyawa M. (1990). Criteria of the classification of spondylarthropathies. Rev. Rhum. Mal. Osteoartic..

[32] Cherkin D.C., Deyo R.A., Volinn E., Loeser J.D. (1992). Use of the International Classification of Diseases (ICD-9-CM) to Identify Hospitalizations for Mechanical Low Back Problems in Administrative Databases. Spine.

[33] Dougados M., van der Linden S., Juhlin R., Huitfeldt B., Amor B., Calin A., Cats A., Dijkmans B., Olivieri I., Pasero G. (1991). The European Spondylarthropathy Study Group Preliminary Criteria for the Classification of Spondylarthropathy. Arthritis Rheum..

[34] Vassar M., Atakpo P., Kash M.J. (2016). Manual Search Approaches Used by Systematic Reviewers in Dermatology. J. Med. Libr. Assoc..

[35] Jovaní V., Blasco-Blasco M., Ruiz-Cantero M.T., Pascual E. (2017). Understanding How the Diagnostic Delay of Spondyloarthritis Differs between Women and Men: A Systematic Review and Metaanalysis. J. Rheumatol..

[36] Calin A., Porta J., Fries J.F., Schurman D.J. (1977). Clinical History as a Screening Test for Ankylosing Spondylitis. Jama.

[37] Aggarwal R., Malaviya A.N. (2009). Diagnosis Delay in Patients with Ankylosing Spondylitis: Factors and Outcomes—An Indian Perspective. Clin. Rheumatol..

[38] Almousa S., Alshamaa N., Wannous H., Khder K., Qasem H. (2023). Gender-Related Differences in Axial Spondyloarthritis (axSpA) Patients. Egypt. Rheumatol..

[39] Aloush V., Ablin J.N., Reitblat T., Caspi D., Elkayam O. (2007). Fibromyalgia in Women with Ankylosing Spondylitis. Rheumatol. Int..

[40] Atagunduz P., Aydin S.Z., Bahadir C., Erer B., Direskeneli H. (2010). Determinants of Early Radiographic Progression in Ankylosing Spondylitis. J. Rheumatol..

[41] Bakland G., Gran J.T., Nossent J.C. (2011). Increased Mortality in Ankylosing Spondylitis Is Related to Disease Activity. Ann. Rheum. Dis..

[42] Bandinelli F., Salvadorini G., Sedie A.D., Riente L., Bombardieri S., Matucci-Cerinic M. (2016). Impact of Gender, Work, and Clinical Presentation on Diagnostic Delay in Italian Patients with Primary Ankylosing Spondylitis. Clin. Rheumatol..

[43] Blasco-Blasco M., Ruiz-Cantero M.T., Juárez-Herrera y Cairo L.A., Jovaní V., Pascual E. (2017). Sex and Gender Interactions in the Lives of Patients with Spondyloarthritis in Spain: A Quantitative-Qualitative Study. J. Rheumatol..

[44] Bodur H., Ataman Ş., Buğdaycı D.S., Rezvani A., Nas K., Uzunca K., Emlakçıoğlu E., Karatepe A.G., Durmuş B., Sezgin M. (2012). Description of the Registry of Patients with Ankylosing Spondylitis in Turkey: TRASD-IP. Rheumatol. Int..

[45] Deodhar A., Mittal M., Reilly P., Bao Y., Manthena S., Anderson J., Joshi A. (2016). Ankylosing Spondylitis Diagnosis in US Patients with Back Pain: Identifying Providers Involved and Factors Associated with Rheumatology Referral Delay. Clin. Rheumatol..

[46] Dincer U., Cakar E., Kiralp M.Z., Dursun H. (2008). Diagnosis Delay in Patients with Ankylosing Spondylitis: Possible Reasons and Proposals for New Diagnostic Criteria. Clin. Rheumatol..

[47] Garrido-Cumbrera M., Navarro-Compan V., Bundy C., Mahapatra R., Makri S., Correa-Fernandez J., Christen L., Delgado-Domínguez C.J., Poddubnyy D., EMAS Working Group (2022). Identifying Parameters Associated with Delayed Diagnosis in Axial Spondyloarthritis: Data from the European Map of Axial Spondyloarthritis. Rheumatology.

[48] Ibn Yacoub Y., Amine B., Laatiris A., Bensabbah R., Hajjaj-Hassouni N. (2012). Relationship between Diagnosis Delay and Disease Features in Moroccan Patients with Ankylosing Spondylitis. Rheumatol. Int..

[49] Landi M., Maldonado-Ficco H., Perez-Alamino R., Maldonado-Cocco J.A., Citera G., Arturi P., Sampaio-Barros P.D., Alvarado D.E.F., Burgos-Vargas R., Santos E. (2016). Gender Differences among Patients with Primary Ankylosing Spondylitis and Spondylitis Associated with Psoriasis and Inflammatory Bowel Disease in an Iberoamerican Spondyloarthritis Cohort. Medicine.

[50] Li J., Xu Y., Chen Y., Ye C., Huang J., Qian L., Xin W., Li T., Ye S. (2019). A Multidisciplinary Clinic Approach to Improve Physician-Related Diagnostic Delay for Patients with Axial Spondyloarthritis: A Retrospective Study. J. Int. Med. Res..

[51] Ma H., Yin Q., Hu F., Guo M., Liu X., Liu Y., Xu Q. (2012). Different Clinical Features in Patients with Ankylosing Spondylitis from Southern and Northern China. Int. J. Rheum. Dis..

[52] Marks S., Barnett M., Calin A. (1983). Ankylosing Spondylitis in Women and Men: A Case-Control Study. J. Rheumatol..

[53] Neuenschwander R., Hebeisen M., Micheroli R., Bürki K., Exer P., Niedermann K., Nissen M.J., Scherer A., Ciurea A. (2020). Differences between Men and Women with Nonradiographic Axial Spondyloarthritis: Clinical Characteristics and Treatment Effectiveness in a Real-Life Prospective Cohort. Arthritis Res. Ther..

[54] Ogdie A., Benjamin Nowell W., Reynolds R., Gavigan K., Venkatachalam S., de la Cruz M., Flood E., Schwartz E.J., Romero B., Park Y. (2019). Real-World Patient Experience on the Path to Diagnosis of Ankylosing Spondylitis. Rheumatol. Ther..

[55] Reed M.D., Dharmage S., Boers A., Martin B.J., Buchanan R.R., Schachna L. (2008). Ankylosing Spondylitis: An Australian Experience. Intern. Med. J..

[56] Ringsdal V.S., Andreasen J.J. (1989). Ankylosing Spondylitis—Experience with a Self Administered Questionnaire: An Analytical Study. Ann. Rheum. Dis..

[57] Roussou E., Sultana S. (2011). Spondyloarthritis in Women: Differences in Disease Onset, Clinical Presentation, and Bath Ankylosing Spondylitis Disease Activity and Functional Indices (BASDAI and BASFI) between Men and Women with Spondyloarthritides. Clin. Rheumatol..

[58] Shahlaee A., Mahmoudi M., Nicknam M.H., Farhadi E., Fallahi S., Jamshidi A.R. (2015). Gender Differences in Iranian Patients with Ankylosing Spondylitis. Clin. Rheumatol..

[59] Slobodin G., Reyhan I., Avshovich N., Balbir-Gurman A., Boulman N., Elias M., Feld J., Mader R., Markovitz D., Rimar D. (2011). Recently Diagnosed Axial Spondyloarthritis: Gender Differences and Factors Related to Delay in Diagnosis. Clin. Rheumatol..

[60] Zink A., Braun J., Listing J., Wollenhaupt J. (2000). Disability and Handicap in Rheumatoid Arthritis and Ankylosing Spondylitis—Results from the German Rheumatological Database. German Collaborative Arthritis Centers. J. Rheumatol..

[61] Higgins J., Li T., Deeks J. (2019). Chapter 6. Section 6.5.2.8: Imputing Standard Deviations for Changes from Baseline. Cochrane Handbook for Systematic Reviews of Interventions Version 6.

[62] von Hippel P.T. (2015). The Heterogeneity Statistic I(2) Can Be Biased in Small Meta-Analyses. BMC Med. Res. Methodol..

[63] Higgins J., Li T., Deeks J. (2019). Chapter 10. Section 10.10.2: Identifying and measuring heterogeneity. Cochrane Handbook for Systematic Reviews of Interventions Version 6.

[64] Fioravanti M., Yanagi M. (2000). Cytidinediphosphocholine (CDP Choline) for Cognitive and Behavioural Disturbances Associated with Chronic Cerebral Disorders in the Elderly. Cochrane Database Syst. Rev..

[65] Khan M.A. (2017). An Update on the Genetic Polymorphism of HLA-B*27 with 213 Alleles Encompassing 160 Subtypes (and Still Counting). Curr. Rheumatol. Rep..

[66] Wu X., Wu J., Li X., Wei Q., Lv Q., Zhang P., Zheng X., Chen Z., Cao S., Tu L. (2020). The Clinical Characteristics of Other HLA-B Types in Chinese Ankylosing Spondylitis Patients. Front. Med..

[67] Mariani F.M., Alunno A., Di Ruscio E., Altieri P., Ferri C., Carubbi F. (2023). Human Leukocyte Antigen B*27-Negative Spondyloarthritis: Clinical, Serological, and Radiological Features of a Single-Center Cohort. Diagnostics.

[68] Paladini F., Fiorillo M.T., Tedeschi V., Cauli A., Mathieu A., Sorrentino R. (2019). Ankylosing Spondylitis: A Trade Off of HLA-B27, ERAP, and Pathogen Interconnections? Focus on Sardinia. Front. Immunol..

[69] Wu X., Wang G., Zhang L., Xu H. (2021). Genetics of Ankylosing Spondylitis-Focusing on the Ethnic Difference Between East Asia and Europe. Front. Genet..

[70] Díaz-Peña R., Castro-Santos P., Durán J., Santiago C., Lucia A. (2020). The Genetics of Spondyloarthritis. J. Pers. Med..

[71] Wang F., Huang S., Gao R., Zhou Y., Lai C., Li Z., Xian W., Qian X., Li Z., Huang Y. (2020). Initial Whole-Genome Sequencing and Analysis of the Host Genetic Contribution to COVID-19 Severity and Susceptibility. Cell Discov..

[72] Wang C.-M., Tan K.-P., Jan Wu Y.-J., Lin J.-C., Zheng J.-W., Yu A.L., Wu J.-M., Chen J.-Y. (2021). MICA*019 Allele and Soluble MICA as Biomarkers for Ankylosing Spondylitis in Taiwanese. J. Pers. Med..

[73] Deodhar A., Gill T., Magrey M. (2023). Human Leukocyte Antigen B27-Negative Axial Spondyloarthritis: What Do We Know?. ACR Open Rheumatol..

[74] Santos M.R., Couto A.R., Foroni I., Bettencourt B.F., Li Z., Meneses R., Wheeler L., Pereira J., Pimentel-Santos F., Fonseca J.E. (2018). Non-Classical Human Leucocyte Antigens in Ankylosing Spondylitis: Possible Association with HLA-E and HLA-F. RMD Open.

[75] Yen Y.-N., Garrido-Cumbrera M., Sun Y.-S., Chen C.-H., Lai C.-C., Tsai H.-C., Chen W.-S., Liao H.-T., Tsao Y.-P., Ankylosing Spondylitis Caring Society of ROC (ASCARES) (2023). The Taiwanese Map of Axial Spondyloarthritis: Living with the Condition. Medicina.

[76] Zuo H., Li M.-M. (2023). Ankylosing Spondylitis and Psychiatric Disorders in European Population: A Mendelian Randomization Study. Front. Immunol..

[77] Yadav S.R.M., Goyal B., Mamgain G., Kothari A., Kumar S., Saha S., Naithani M., Mirza A.A., Kumar R., Arora R. (2023). Genetic Variations in IL-1β, TNF-α, and TGF-β Associated with the Severity of Chronic Cervical Spondylitis in Patients. Cells.

[78] Canossi A., Oumhani K., Del Beato T., Sebastiani P., Colanardi A., Aureli A. (2023). The Role of CD1 Gene Polymorphism in the Genetic Susceptibility to Spondyloarthropathies in the Moroccan Population and the Possible Cross-Link with Celiac Disease. Vaccines.

[79] Mathieu A., Cauli A., Fiorillo M.T., Sorrentino R. (2008). HLA-B27 and Ankylosing Spondylitis Geographic Distribution as the Result of a Genetic Selection Induced by Malaria Endemic? A Review Supporting the Hypothesis. Autoimmun. Rev..

[80] Bugaj B., Wielińska J., Bogunia-Kubik K., Świerkot J. (2022). Searching for New Genetic Biomarkers of Axial Spondyloarthritis. J. Clin. Med..

