# Supplementary material for: Sex Bias in Diagnostic Delay: Are Axial Spondyloarthritis and Ankylosing Spondylitis Still Phantom Diseases in Women? A Systematic Review and Meta-Analysis
Source: J Pers Med. 2024 Jan 13;14(1):91. doi: 10.3390/jpm14010091 (PMC10817445; doi:10.3390/jpm14010091)
Supplement: Supplementary file 1 [file jpm-14-00091-s001.zip › jpm-2815782-supplementary.pdf]

**Table S1. Studies selected**	**Selection F vs M**	**Comparability**	**DD ascertainment**	**m-NOS (0-8)**
**Aggarwal 2009**	3	0	2	5 medium-high
**Almousa 2023**	3	1	2	6 high
**Aloush 2007**	3	0	2	5 medium-high
**Atagunduz 2010**	4	1	2	7 high
**Bakland 2011**	4	1	2	7 high
**Bandinelli 2016**	4	0	2	6 high
**Blasco Blasco 2017**	2	1	2	5 medium-high
**Bodur 2012**	4	0	2	6 high
**Dehodar 2016**	1	0	2	3 low
**Dincer 2008**	4	1	2	7 high
**Garrido-cumbrera 2021**	4	0	2	6 high
**Hajialilo 2014**	4	0	2	6 high
**Ibn Yacoub 2012**	4	1	2	7 high
**Jovani 2018**	4	1	2	7 high
**Landi 2016**	4	0	2	6 high
**Li 2019**	4	1	2	7 high
**Ma 2012**	3	1	2	6 high
**Marks 1983**	1	1	2	4 medium
**Neunshwander 2020**	4	1	2	7 high
**Ogdie 2019**	1	0	2	3 low
**Reed 2008**	3	0	2	5 medium-high
**Ringsdal 1989**	1	1	2	4 medium
**Roussou 2011**	1	1	2	4 medium
**Shahlaee A 2015**	3	1	3	7 high
**Slobodin 2011**	4	1	1	6 high
**Zink 2000**	4	1	2	7 high
Selection (0-4): female (F) disease definition and representativeness, men (M) selection and disease definition; Sexes comparability (0-1); Ascertainment of diagnostic delay (DD) (records 0-1, same method between sexes 0-1, not response rate 0-1 =0-3). Abbreviation: m-NOS: modified newcastle-ottawa quality assessment scale.				

Table S1. m-NOS quality assessment of 26 studies eligible of systematic review

A.

	MEAN DIFFERENCE	95% CI	WEIGHT	V	SE	SIG.	TOTAL	WOMEN	MEN
Aggarwal 2009	2.10	-1.98 / 6.18	14.80%	4.33	2.08	0.313	70	11	59
Almousa 2023	2.50	1.01 / 3.99	39.60%	0.58	0.76	0.001	104	38	66
Ibn Yaccoub 2012	0.20	-0.84 / 1.24	45.60%	0.28	0.53	0.705	130	43	87
Overall (random-effects model)	1.39	-0.46 / 3.24	100.00%	0.89	0.94	0.140	304	92	212

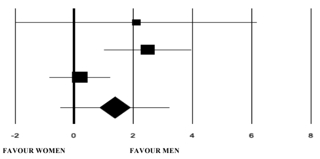

CHI^2 6.48 I^2 69.11 DF 2 P= 0.039

MEAN DIAGNOSTIC DELAY IN WOMEN MINUS MEAN DIAGNOSTIC DELAY IN MEN

B.

	MEAN DIFFERENCE	95% CI	WEIGHT	V	SE	SIG.	TOTAL	WOMEN	MEN
Aloush 2007	5.80	0.44 / 11.16	4.49%	7.47	2.73	0.034	36	18	18
Atagunduz 2010	1.20	-0.77 / 3.17	7.35%	1.01	1.01	0.233	235	96	139
Bandinelli 2016	-3.57	-3.94 / -3.20	8.13%	0.04	0.19	0.000	135	44	91
Bodour 2012	0.40	-0.45 / 1.25	8.00%	0.19	0.43	0.357	1381	343	1038
Dincer 2008	9.10	-0.83 / 19.03	2.14%	25.66	5.07	0.072	111	8	103
Garrido-Cumbrera 2021	2.10	1.50 / 2.70	8.08%	0.10	0.31	0.000	2846	1746	1100
Hajialilo 2014	2.10	-1.49 / 5.69	5.97%	3.36	1.83	0.252	60	7	53
Landi 2016	-1.10	-2.18 / -0.02	7.90%	0.30	0.55	0.046	1072	255	817
Marks 1983	2.50	-4.33 / 9.33	3.50%	12.14	3.48	0.473	26	18	8
Neuenschwander 2020	1.30	-0.06 / 2.66	7.76%	0.48	0.69	0.061	495	264	231
Reed 2008	2.90	0.13 / 5.67	6.70%	1.99	1.41	0.040	126	35	91
Ringsdal 1989	3.10	1.85 / 4.35	7.82%	0.41	0.64	0.000	179	48	131
Roussou 2011	0.71	-0.80 / 2.22	7.67%	0.59	0.77	0.357	443	293	150
Shahlaee 2015	0.80	-1.42 / 3.02	7.16%	1.29	1.13	0.481	320	67	253
Slobodin 2011	-0.20	-2.18 / 1.78	7.34%	1.02	1.01	0.843	151	72	79
Overall (random-effects model)	1.21	-0.48 / 2.91	100.00%	0.74	0.86	0.159	7616	3314	4302

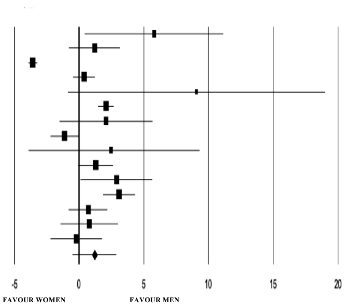

CHI^2 383.99 I^2 96.35 DF 14 P= 0.000

MEAN DIAGNOSTIC DELAY IN WOMEN MINUS MEAN DIAGNOSTIC DELAY IN MEN

Figure S1: Forest plot of the differences between the mean diagnosis delay of Ax-SpA and AS in women versus men according to World Bank economic class in: A. papers from low and lower-middle income countries; B. papers from upper-middle- and high-income countries.

A.

	MEAN DIFFERENCE	95% CI	WEIGHT	V	SE	SIG.	TOTAL	WOMEN	MEN
Aggarwal 2009	2.10	-1.98 / 6.18	5.24%	4.33	2.08	0.313	70	11	59
Aloush 2007	5.80	0.44 / 11.16	3.13%	7.47	2.73	0.034	36	18	18
Dincer 2008	9.10	-0.83 / 19.03	0.94%	25.66	5.07	0.072	111	8	103
Hajialilo 2014	2.10	-1.49 / 5.69	6.62%	3.36	1.83	0.252	60	7	53
Ibn Yaccoub 2012	0.20	-0.84 / 1.24	40.41%	0.28	0.53	0.705	130	43	87
Marks 1983	2.50	-4.33 / 9.33	1.96%	12.14	3.48	0.473	26	18	8
Roussou 2011	0.71	-0.80 / 2.22	26.57%	0.59	0.77	0.357	443	293	150
Shahlaee 2015	0.80	-1.42 / 3.02	15.14%	1.29	1.13	0.481	320	67	253
Overall (random-effects model)	0.96	-0.01 / 1.92	100.00%	0.24	0.49	0.053	1196	465	731

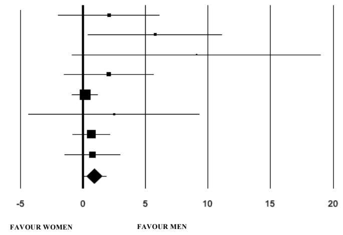

CHI^2 8.43 I^2 16.94% DF 7 P 0.300

MEAN DIAGNOSTIC DELAY IN WOMEN MINUS MEAN DIAGNOSTIC DELAY IN MEN

B.

	MEAN DIFFERENCE	95% CI	WEIGHT	V	SE	SIG.	TOTAL	WOMEN	MEN
Almousa 2023	2.50	1.01 / 3.99	10.01%	0.58	0.76	0.001	104	38	66
Atagunduz 2010	1.20	-0.77 / 3.17	9.59%	1.01	1.01	0.233	235	96	139
Bandinelli 2016	-3.57	-3.94 / -3.20	10.57%	0.04	0.19	0.000	135	44	91
Bodour 2012	0.40	-0.45 / 1.25	10.40%	0.19	0.43	0.357	1381	343	1038
Garrido-Cumbrera 2021	2.10	1.50 / 2.70	10.50%	0.10	0.31	0.000	2846	1746	1100
Landi 2016	-1.10	-2.18 / -0.02	10.28%	0.30	0.55	0.046	1072	255	817
Neuenschwander 2020	1.30	-0.06 / 2.66	10.10%	0.48	0.69	0.061	495	264	231
Reed 2008	2.90	0.13 / 5.67	8.78%	1.99	1.41	0.040	126	35	91
Ringsdal 1989	3.10	1.85 / 4.35	10.18%	0.41	0.64	0.000	179	48	131
Slobodin 2011	-0.20	-2.18 / 1.78	9.59%	1.02	1.01	0.843	151	72	79
Overall (random-effects model)	0.82	-1.16 / 2.80	100.00%	1.02	1.01	0.417	6724	2941	3783

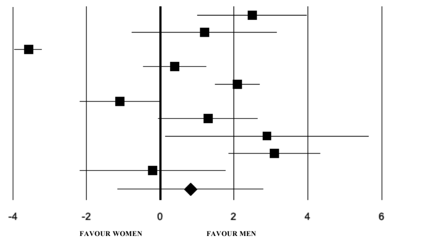

CHI^2 383.63 I2 97.65% DF 9 P 0.000

MEAN DIAGNOSTIC DELAY IN WOMEN MINUS MEAN DIAGNOSTIC DELAY IN MEN

Figure S2: Forest plot of the differences between the mean diagnosis delay of of Ax-SpA and AS in women versus men according to samples of:

A. mono-centric B multi-centric papers.
